# Association between prevalence rate of dementia with Lewy bodies and sleep characteristics in Chinese old adults

**DOI:** 10.3389/fnhum.2022.976753

**Published:** 2022-09-14

**Authors:** Jinghuan Gan, Shuai Liu, Fei Wang, Zhihong Shi, Yang Lü, Jianping Niu, Xinling Meng, Pan Cai, Xiao-Dan Wang, Zhichao Chen, Baozhi Gang, Yong Ji

**Affiliations:** ^1^Department of Neurology, Beijing Tiantan Hospital, Capital Medical University, Beijing, China; ^2^Department of Neurology, Tianjin Dementia Institute, Tianjin Key Laboratory of Cerebrovascular and Neurodegenerative Diseases, Tianjin Huanhu Hospital, Tianjin, China; ^3^Department of Neurology, Yuncheng Central Hospital of Shanxi Province, Shanxi, China; ^4^Department of Geriatrics, The First Affiliated Hospital of Chongqing Medical University, Chongqing, China; ^5^Department of Neurology, The Second Affiliated Hospital of Xiamen Medical College, Xiamen, China; ^6^Department of Neurology, Affiliated Traditional Chinese Medicine Hospital of Xinjiang Medical University, Urumqi, China; ^7^Dementia Clinic, Affiliated Hospital of Zunyi Medical University, Zunyi, China; ^8^Department of Neurology, The First Affiliated Hospital of Harbin Medical University, Harbin, China

**Keywords:** sleep duration, dementia, cognitive impairment, dementia with Lewy body, Parkinsonism

## Abstract

**Introduction:** Few studies are available on the prevalence and sleep-related factors of dementia with Lewy bodies (DLB) in Chinese older adults, aiming to explore the associations between sleep characteristics and DLB.

**Methods:** A cross-sectional study with 7,528 individuals aged ≥65 years in 106 communities in Northern China was conducted from April 2019 to January 2020. Questionaries (including demographic characteristics, comorbidities, lifestyles, and sleep characteristics) were administered, and neuropsychological assessments and physical examination were conducted in phase I; screening for probable DLB was done in phase II. Logistic regressions were used to assess associations.

**Results:** A total of 919 (12.2%, 919/7,528) participants had dementia, and 101 (1.3%, 101/7,528) participants were diagnosed with DLB. The prevalence of dementia and DLB were slightly higher or equal in women, increased with age, and roughly decreased with nighttime sleep duration. Of the 101 participants, all of them (100.0%) had cognitive impairment, 46 (44.54%) displayed fluctuating cognition, 72 (71.29%) of them showed visual hallucination, 22 (21.78%) individuals reported RBD, and 27.71% showed Parkinsonism. Sleeping for <5 h (adjusted OR = 1.795, 95%CI: 1.055–3.054, *p* < 0.05) or having hypersomnolence (adjusted OR = 31.213, 95% CI: 17.618–55.301, *p* < 0.001) were significantly associated with the occurrence of DLB. Sleep duration of <5 h or >8 h had combined diagnostic value for DLB (AUC = 0.783, 95%CI: 0.734–0.831, *p* < 0.001).

**Conclusions:** The current prevalence of DLB is 1.3% in Northern China. Short or long nighttime sleep duration is independently associated with the occurrence of dementia and DLB.

## Introduction

In recent years, dementia has been expected to become prevalent in the rapidly aging population. Mounting evidence shows that abnormal sleep duration is related to cognitive decline among older adults. Specifically, the J-shaped, U-shaped or even V-shaped non-linear relationship of sleep duration with the risks of dementia has been proved (Liang et al., 2019), where either long or short sleep duration could increase dementia risk in old age (Ramos et al., 2020).

Several studies have indicated that sleep is a promising modifiable risk factor for Alzheimer’s disease (AD). Pathological tau and β-amyloid (Aβ) aggregation are the hallmark features of AD in both mice and humans and has a close relationship to nonrapid eye movement and sleep disruption in early AD. Non-linear associations of cerebrospinal fluid (CSF) alpha-synuclein with sleep duration were revealed in a cohort study (Wang et al., 2020), and poor sleep quality and excessive or insufficient sleep were associated with lower levels of CSF alpha-synuclein. Systematic reviews also have demonstrated that patients with Parkinson’s disease (PD), a neurodegenerative disease characterized by rigidity, tremor, and bradykinesia, have poor sleep quality and quantity, short sleep duration, indicative of impairments in cognitive function (Zhang et al., 2020). On the basis of current research and diagnostic criteria for dementia with Lewy bodies (DLB; McKeith et al., 2017), sleep disorders are commonly regarded as diagnosable features for DLB. For example, rapid eye movement sleep behavior disorder (RBD) is a core clinical feature, and hypersomnia is a supportive clinical feature. Notably, few studies have examined the association between sleep duration and DLB in large-sample studies of older adults.

To the best of our knowledge, there is limited information available about the clinical features of DLB in Chinese rural older adults in a large sample. Thus, we conducted a population-based cross-sectional survey to reveal the core clinical features of DLB in the general population and then to analyze the associations between sleep duration and the risks of dementia and its subtypes, exploring the possible combined diagnostic value of sleep duration for dementia and DLB.

## Material and Methods

### Participants

This cross-sectional study enrolled participants ≥65 years of age in 106 community primary health care centers selected from 949 villages in rural Ji County in Northern China between April 2019 and January 2020, and it was aimed at investigating the associations between sleep and dementia. The local medical practitioner in each village (who had worked there for over 5 years) was involved in identifying all individuals aged ≥65 years based on the date of birth provided on the residence certificate.

**Figure 1:**
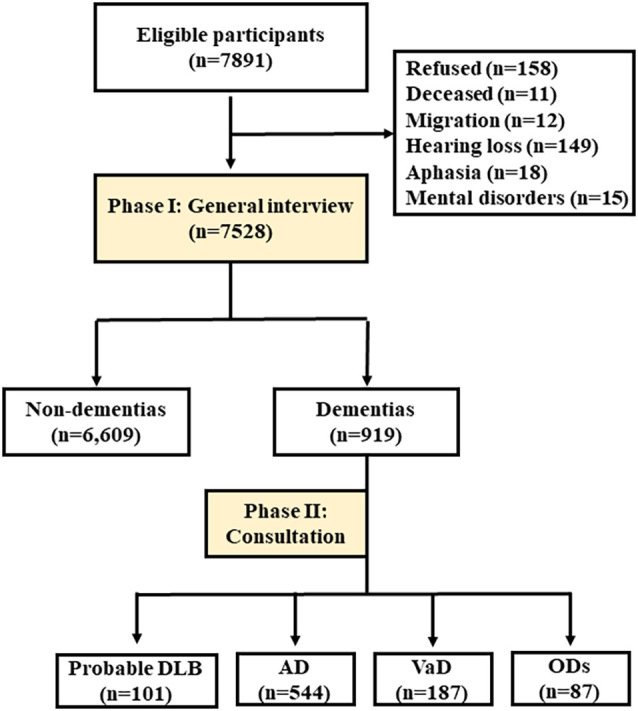
Flow chart of this study. The flow chart of the survey with two phases was showed, to investigate the prevalence rates of dementia and its subtypes. DLB, dementia with Lewy bodies; AD, Alzheimer’s disease; VaD, vascular dementia; ODs, other dementia.

Figure 1 shows the flow chart of study enrollment and exclusion. The total number of participants aged ≥65 years in these communities was 7,891; however, due to refusal (*n* = 158), death (*n* = 11), migration (*n* = 12), hearing loss (*n* = 149), aphasia (*n* = 18), or mental disorders (*n* = 15), a total of 7,528 completed the final interview. The study was approved by the Committee for Medical Research Ethics at Tianjin Huanhu Hospital and the Tianjin Health Bureau (ID: 2019-40). Written informed consent was obtained from each subject either directly or from their guardian.

### Measures

A door-to-door, face-to-face questionnaire-based survey was conducted by senior medical students or medical staff in the local panel health centers using two phases. A neurologist with special expertise in cognitive impairment and Parkinsonism syndrome re-reviewed the data in each region. All interviewers and experts received the same one-week training on collecting information (including basic demographic characteristics: gender, age, education, marriage, and occupation; comorbidities: stroke, diabetes mellitus (DM), heart disease, and hypertension; lifestyles: smoking and alcohol consumption; sleep characteristics: self-reported nighttime sleep duration and sleep disorders); and neuropsychological assessments and diagnosis. In addition, they participated in a retraining course every 2 months.

Age was classified as 65–69, 70–74, 75–79, 80–84, and ≥85 years of age. Educational attainment was divided into three levels: 0, 1–6, and more than 6 years of education. The single, widowed, or divorced participants were recorded as “unmarried”. Those living with children, friends, or relatives, or at a nursing home were recorded as living “with others”. None of the participants included in the analysis lived alone. A smoker was defined as an individual with a history of smoking ≥5 cigarettes per day for >2 years. An alcohol drinker was defined as an individual with a history of drinking an alcoholic beverage ≥1 time per week for >2 years. Self-reported nighttime sleep duration in last month was divided into five categories: 1–2 h, 3–4 h, 5–6 h, 7–8 h, and >8 h. “Hallucinations in other modalities” included “auditory hallucination”, “tactile hallucination”, “olfactory hallucination”, and other types of hallucinations except for visual hallucination.

#### Phase I: general interview

A centralized medical examination at the health station and an in-person survey were conducted. All information was collected *via* questionnaires. Each participant then underwent a neurological examination by senior medical students or medical staff.

The Pittsburgh Sleep Quality Index (Buysse et al., 1989) was used to evaluate sleep quality and disturbances during the last month. RBD and hypersomnolence were assessed by the rapid eye movement sleep behavior disorder screening questionnaire and Epworth Sleepiness Scale, respectively. Fluctuations including spontaneous alterations in cognition, attention, and arousal were rated positive according to the participant’s or caregiver’s complaints of changing during the day and over weeks, and then assessed using the One Day Fluctuation Scale and the Clinical Assessment of Fluctuation scale (Walker et al., 2000). Participants with cardinal features of Parkinsonism were defined as having one or more spontaneous bradykinesia (defined as slowness of movement and decrement in amplitude or speed), rest tremor, or rigidity after the neurological examination. Participants with a history of diagnosis and treatment of PD were included as well. Psychiatric systems such as hallucinations, anxiety, and depression were assessed by neuropsychiatric inventory (NPI; Cummings et al., 1994), which was conducted with their caregivers (children, spouse, staff nurse, or friend). And scores ≥1 on the NPI-hallucination subitem were recorded as “presence of hallucination”. The chief complaint of seeing people, children, or animals that were not present was recorded as “visual hallucination”, and hearing things that were not present was recorded as “auditory hallucination”; feeling something on the skin or seeming to feel something crawling or touching on the body was recorded as “tactile hallucination”; smelling something that was not present was recorded as “olfactory hallucination”. The autonomic nervous system (ANS) function was assessed by Scales for Outcomes in Parkinson’s Disease-Autonomic questionnaire (Hattori et al., 2020). The evaluations for cognitive performance were the Mini-Mental State Examination-Chinese version (C-MMSE; Folstein et al., 1975), the Clinical Dementia Rating (CDR) scale (Morris, 1993), and the Activities of Daily Living (ADL) scale (Eto et al., 1992).

Participants with C-MMSE score below the cutoff points 17, 20, and 24 points for those who were illiterate or had only gone to primary school or had higher education, respectively (Zhang et al., 1990), and/or a CDR of 0.5 or higher, were deemed eligible for phase II of the study.

#### Phase II: consultation

##### For all-cause dementia and DLB

Participants who were eligible for phase II of the study were examined for the presence of all-cause dementia and main subtypes of dementia (particularly “probable DLB”). Physical examination, blood tests (thyroid function, syphilis, HIV, and vitamin B12 level), and a neuroimaging examination [magnetic resonance imaging (MRI) or computed tomography, if the participant could not undergo MRI] were arranged to obtain or rule out a diagnosis of dementia. ^11^C-PIB PET scan (*n* = 9) and ^18^F-FDG PET scan (*n* = 17) were used if possible for those difficult to diagnose. All diagnoses were confirmed blindly by two board-certified neurologists and, if there was disagreement between them, the subject was excluded.

To distinguish DLB from Parkinson’s disease dementia (PDD), we excluded patients in whom cognitive impairment had occurred more than 1 year after they were diagnosed with the extrapyramidal syndrome. The clinical symptoms and signs of the DLBs were also collected.

### Diagnostic criteria

In this study, we diagnosed dementia on the basis of the Diagnostic and Statistical Manual of Mental Disorders IV (DSM-IV) criteria (American Psychiatric Association, 1994). The National Institute of Neurological and Communicative Disorders-AD and Related Disorders Association criteria were used for the clinical diagnosis of AD (McKhann et al., 2011). We also used the National Institute of Neurological Disorders and Stroke–Association Internationale pour la Recherche et l’Enseignementen Neurosciences for vascular dementia (VaD; Román et al., 1993). The diagnosis of probable DLB was conducted by using consensus criteria for DLB established in 2017 by McKeith et al. (2017) with two or more core clinical features of DLB present, with or without the presence of indicative biomarkers. Other dementias (ODs) defined by globally accepted criteria included mixed dementia, frontotemporal lobe dementia (FTLD), PDD, alcoholic dementia, hydrocephalus dementia, and posttraumatic dementia.

### Data analysis

The prevalence rates of dementia and subtypes were estimated for the entire study population and according to 5-year age group, gender, and sleep duration as well. Frequency distributions were used to analyze the qualitative variables. We calculated descriptive statistics for continuous variables. The Student’s t-test was used for continuous variables consistent with normal distribution and nonparametric test for non-normal applications. The chi-square and Mann-Whitney U tests were used to assess the clinical factors associated with the presence of dementia.

We performed crude and adjusted regression analyses with sleep duration/sleep disorders as the covariates and dementia/DLB as the outcome. Logistic regression models were used to estimate the odds ratios (ORs) for each outcome associated with self-reported sleep duration, adjusting for potential confounders. In this study, 5–8 h per night was modeled as the reference category (Ref.) in regression analysis. Covariates associated with self-reported sleep duration and at the *p* < 0.10 level were included in the models, and sex and age were also included for face validity. All data were analyzed using IBM SPSS Statistics for Windows (Version 25.0; IBM Corp., Armonk, NY, USA). p-values < 0.05 were considered statistically significant.

## Results

### Dementia characteristics in samples

**Figure 2:**
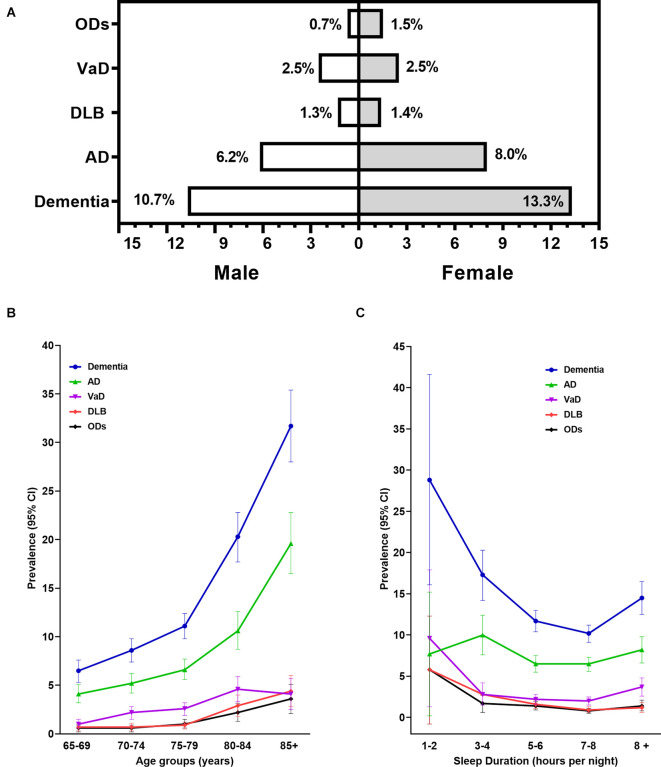
Prevalence of all-cause dementia and main subtypes by age, gender, and sleep duration categories. Panel **(A)** showed the crude prevalence rates of dementia and its subtypes. Panels **(B,C)** showed the prevalence and 95% CI of dementia and its subtypes by age and sleep duration categories. 95% CI, 95% confidence interval; AD, Alzheimer’s disease; VaD, vascular dementia; DLB, dementia with Lewy bodies; ODs, other dementia.

Overall, 7,528 participants aged ≥65 years old completed all survey documents (Table 1), of whom 919 (12.2%) had dementia: 544 (7.2%) with AD, 187 (2.5%) with VaD, 101 (1.3%) with DLB, and 87 (1.2%) with ODs. The prevalence of dementia was higher in women (13.3% *vs*. 10.7%, shown in Figure 2A), increased with age, from 6.5% (95%CI: 5.3%–7.6%) at ages 65–69 years to 31.7% (95%CI: 28.0%–35.4%) at ages 85 years or older (Figure 2B), and was significantly higher in participants with <5 h or >8 h of sleep per night (Figure 2C). The prevalence of DLB increased with age, from 0.7% (95%CI: 0.3%–1.1%) to 4.4% (95%CI: 2.8%–6.0%), and decreased with nighttime sleep duration prolonging to 7–8 h per night, from 5.8% (95%CI: 0.8%–12.3% at 1–2 h per night) to 0.9% (95%CI: 0.6%–1.3% at 7–8 h per night).

**Table 1:** Sample characteristics by cognitive performance.

	**All samples**		**Non-dementia**		**Dementia**		**DLB**		**P1-value**	**P2-value**
	**Num.**	**Pro. (%)**	**Num.**	**Pro. (%)**	**Num.**	**Pro. (%)**	**Num.**	**Pro. (%)**		
**Num. of samples**	7,528		6,609		919		101	
**Age (years)**	74.85 ± 6.29		74.33 ± 5.94		78.61 ± 7.42		80.24 ± 8.14		0.000	0.000
65–69	1,746	23.2	1,633	24.7	113	12.3	13	12.9		
70–74	1,988	26.4	1,817	27.5	171	18.6	14	13.9		
75–79	2,220	29.5	1,974	29.9	246	26.8	19	18.8		
80–84	962	12.8	767	11.6	195	21.2	28	27.7		
≥85	612	8.1	418	6.3	194	21.1	27	26.7		
**Gender**									0.001	0.731
Male	3,204	42.6	2,861	43.3	343	37.3	42	41.6		
Female	4,324	57.4	3,748	56.7	576	62.7	59	58.4		
**Education (years)**	5.73 ± 4.51		5.96 ± 4.49		4.10 ± 4.30		4.61 ± 4.95		0.000	0.000
< 1	1,489	19.8	1,187	18.0	302	32.9	31	30.7		
1–6	3,173	42.1	2,786	42.2	387	42.1	44	43.6		
>6	2,866	38.1	2,636	39.9	230	25.0	26	25.7		
**Marriage**									0.000	0.000
Married	5,747	76.3	5,171	78.2	576	62.7	54	53.5		
Un-married	1,781	23.7	1,438	21.8	343	37.3	47	46.5		
**Living status**									0.000	0.000
With spouse	5,133	68.2	4,641	70.2	492	53.5	41	40.6		
Others	2,395	31.8	1,968	29.8	427	46.5	60	59.4		
**Occupation**									0.000	0.384
Non-manual	3,479	46.2	3,116	47.1	363	39.5	52	51.5		
Manual	4,049	53.8	3,493	52.9	556	60.5	49	48.5		
**Smoking**	1,815	24.1	1,623	24.6	192	20.9	29	28.7	0.015	0.336
**Alcohol consumption**	1,568	20.8	1,415	21.4	153	16.6	21	20.8	0.001	0.881
**Stroke**	840	11.2	666	10.1	174	18.9	17	16.8	0.000	0.026
**DM**	1,142	15.2	1,003	15.2	139	15.1	22	21.8	0.968	0.067
**Heart disease**	1,211	16.1	1,053	15.9	158	17.2	24	23.8	0.330	0.033
**Hypertension**	3,843	51.0	3,371	51.0	472	51.4	54	53.5	0.841	0.624
**Parkinsonism syndrome**										
Rest tremor	271	3.6	223	3.4	48	5.2	37	36.6	0.005	0.000
Rigidity	103	1.4	85	1.3	18	2.0	18	17.8	0.100	0.000
Bradykinesia	255	3.4	211	3.2	44	4.8	43	42.6	0.012	0.000
Postural instability	25	0.3	23	0.3	2	0.2	2	2.0	0.520	0.008
**ANS dysfunction symptoms**										
OH	763	10.1	672	10.2	91	9.9	23	22.8	0.802	0.000
Sexual dysfunction	267	3.5	235	3.6	32	3.5	11	10.9	0.910	0.000
HH	1,020	13.5	903	13.7	117	12.7	34	33.7	0.439	0.000
Constipation	1,144	15.2	988	14.9	156	17.0	32	31.7	0.109	0.000
Urinary incontinence	1,161	15.4	1,023	15.5	138	15.0	31	30.7	0.716	0.000
**Sleep disorders**										
Hypersomnia	101	1.3	73	1.1	28	3.0	28	27.7	0.000	0.000
Insomnia	1,804	24.0	1,577	23.9	227	24.7	30	29.7	0.576	0.172
RBD	201	2.7	167	2.5	34	3.7	22	21.8	0.039	0.000
**Current night-sleep duration (hours)**									0.000	0.000
< 5	654	8.7	535	8.1	119	12.9	20	19.8		
5–8	5,588	74.2	4,986	75.4	602	65.5	67	66.3		
>8	1,195	15.9	1,022	15.5	173	18.8	14	13.9		
Missed	91	1.2	66	1.0	25	2.7	0.0	0.0		
**C-MMSE score**	25.02 ± 5.28		26.46 ± 3.18		14.61 ± 5.67		14.56 ± 5.89		0.000	0.000
**ADL score**	22.22 ± 6.11		20.42 ± 0.97		35.21 ± 10.34		37.24 ± 10.23		0.000	0.000
**CDR score**	0.21 ± 0.63		‥0.00 ± 0.00		1.76 ± 0.72		1.77 ± 0.71		0.000	0.000
0–0.5	6,609	87.8	6,609	100.0	–	–	–	–		
1.0	375	5.0	–	–	375	40.8	39	38.6		
2.0	392	5.2	–	–	392	42.7	46	45.6		
3.0	152	2.0	–	–	152	16.5	16	15.8		

P1, dementia vs. non-dementia; P2, DLB vs. non-dementia; DLB, dementia with Lewy bodies; Num., Number; Pro., proportion; DM, diabetes mellitus; ANS dysfunction symptoms, autonomic nervous system dysfunction symptoms; OH, orthostatic hypotension; HH, hyperhidrosis; RBD, rapid eye movement sleep behavior disorder; C-MMSE, the Mini-Mental State Examination (Chinese version); ADL, the Activity of Daily Living Scale; CDR, the clinical dementia rating.

### Clinical features of DLB

The clinical features of the Chinese population with DLB were recorded (Table 2). Of the 101 participants, 46 (44.54%) displayed fluctuating cognition. All the participants (100.0%) had cognitive impairment, while 72 (71.29%) of them showed visual hallucination. Twenty-two (21.78%) individuals reported RBD, and 27.71% showed Parkinsonism. The supportive clinical features occurring in the Chinese DLBs were analyzed. The elder population with DLB suffered a proportion of repeated falls (12.87%), hypersomnia (27.72%), systematized delusions (25.74%), apathy (24.75%), anxiety (18.81%), or depression (9.9%). Moreover, the DLBs displayed autonomic dysfunction, including orthostatic hypotension (22.77%), sexual dysfunction (10.89%), hyperhidrosis (33.66%), constipation (31.68%), and urinary incontinence (30.69%).

**Table 2:** Clinical features of Chinese population with DLB.

	**Num. of Cases**	**Pro. (%)**
**All DLB samples**	101	100.00%
**Essential**		
Cognitive impairment	101	100.00%
**Core clinical features**		
Fluctuating cognition	46	44.54%
Parkinsonism (bradykinesia, rest tremor, or rigidity)	29	27.71%
Visual hallucination	72	71.29%
RBD	22	21.78%
**Supportive clinical features**		
Severe sensitivity to antipsychotic agents	5	5.00%
Postural instability	2	1.98%
Repeated falls	13	12.87%
Syncope or other transient episodes of unresponsiveness	4	3.96%
Severe autonomic dysfunction		
OH	23	22.77%
Sexual dysfunction	11	10.89%
HH	34	33.66%
Constipation	32	31.68%
Urinary incontinence	31	30.69%
Hypersomnia	28	27.72%
Hyposmia	6	5.94%
Hallucinations in other modalities	5	5.00%
Systematized delusions	26	25.74%
Apathy	25	24.75%
Anxiety	19	18.81%
Depression	10	9.90%

DLB, dementia with Lewy bodies; Num., Number; Pro., proportion; OH, orthostatic hypotension; HH, hyperhidrosis; RBD, rapid eye movement sleep behavior disorder.

**Figure 3:**
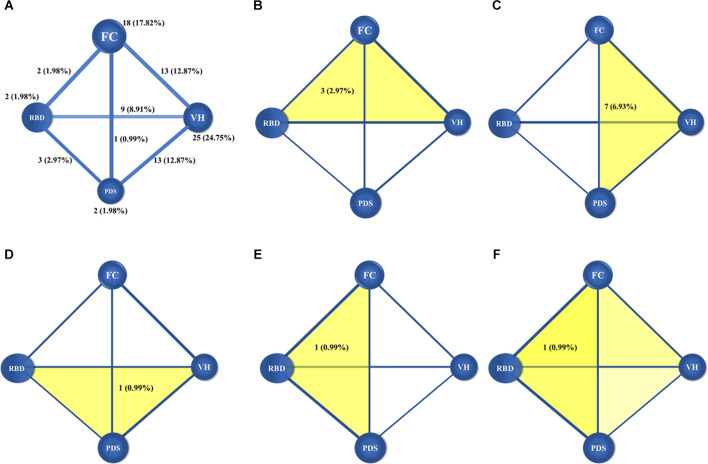
Figures showed the number and proportions of DLB cases with one and two **(A)** core clinical features, three core clinical features **(B–E)** and four core clinical features **(F)**. The balls meant the core clinical features, and the lines and yellow patterns meant the combinations of core clinical features. In panel **(A)**, the numbers and proportions in the balls meant the distributions of individual core clinical feature, and the numbers and proportions in the lines meant the combinations of any two core clinical features. In panels **(B–E)**, the numbers and proportions in the yellow patterns meant the combinations of any three core clinical features, and in panel **(F)**, the number and proportion in the yellow patterns meant the combinations of four core clinical features. DLB, dementia with Lewy bodies; FC, fluctuating cognition; PDS, Parkinsonism; VH, visual hallucination; RBD, rapid eye movement sleep behavior disorder.

In addition, the number and proportions of cases that shared specific core features within the data are shown in Figure 3. Eighteen participants (17.82%) displayed only fluctuating cognition, two (1.98%) displayed only Parkinsonism, 25 displayed (24.75%) only visual hallucinations, and two (1.98%) reported RBD. Thirteen participants (12.87%) displayed fluctuating cognition and visual hallucinations, nine participants (8.91%) displayed visual hallucinations and RBD, 13 participants (12.87%) displayed visual hallucinations and Parkinsonism, two (1.98%) displayed fluctuating cognition and RBD, one (0.98%) displayed fluctuating cognition and Parkinsonism, and three (2.97%) displayed RBD and Parkinsonism. Three participants (2.97%) displayed visual hallucinations, fluctuating cognition, and RBD, seven participants (6.93%) displayed visual hallucinations, fluctuating cognition, and Parkinsonism, one person (0.99%) displayed visual hallucinations, RBD, and Parkinsonism, and another one (0.99%) displayed fluctuating cognition, RBD, and Parkinsonism. Only one person (0.99%) displayed all four core features.

### Sleep and dementia

We evaluated the association between sleep and the risk of dementia (Table 3). Relative to participants sleeping 5–8 h in current stage, those with <5 h sleep duration were 1.842 (1.485–2.286) times more likely to have dementia, and a similar result was obtained for those with >8 h (OR = 1.402, 95%CI: 1.169–1.682, *p* < 0.05). We also found that sleep for <5 h significantly increased risk of DLB around two times (OR = 2.126, 95%CI: 1.156–3.908 in crude model; OR = 1.795, 95% CI: 1.055–3.054 in adjusted model, *p* < 0.05).

**Table 3:** Logistic regression analysis for dementia by sleep characteristics.

		**Current night-sleep duration (OR, 95% CI)**			**Sleep disorders (OR, 95% CI)**		
	**< 5 h**	**5–8 h (Ref.)**	**>8 h**	**RBD**	**Hypersomnolence**	**Insomnia**
**Dementia**	**Crude**	1.842 (1.485–2.286)**	1	1.402 (1.169–1.682)**	1.482 (1.018–2.157)*	2.814 (1.810–4.374)**	1.047 (0.892–1.229)
	**Adjusted**	1.299 (1.032–1.635)*	1	1.257 (1.038–1.522)*	1.162 (0.779–1.732)	2.458 (1.519–3.978)**	0.983 (0.829–1.167)
**AD**	**Crude**	1.648 (1.245–2.181)**	1	1.321 (1.046–1.667)*	0.503 (0.235–1.077)	None	0.982 (0.799–1.206)
	**Adjusted**	1.288 (0.960–1.730)	1	1.210 (0.947–1.546)	0.464 (0.213–1.012)	None	0.986 (0.794–1.224)
**VaD**	**Crude**	1.768 (1.111–2.812)*	1	1.851 (1.300–2.635)*	0.417 (0.103–1.694)	None	1.041 (0.743–1.459)
	**Adjusted**	0.962 (0.563–1.643)	1	1.508 (1.007–2.258)*	0.241 (0.055–1.043)	None	0.881 (0.599–1.296)
**DLB**	**Crude**	2.126 (1.156–3.908)*	1	1.455 (0.843–2.511)	10.965 (6.668–18.031)**	35.677 (27.742–58.543)**	1.368 (0.889–2.104)
	**Adjusted**	1.795 (1.055–3.054)*	1	0.891 (0.492–1.614)	9.510 (5.521–16.383)**	31.213 (17.618–55.301)**	1.239 (0.791–1.939)
**ODs**	**Crude**	2.782 (1.675–4.620)**	1	1.019 (0.571–1.821)	1.378 (0.431–4.403)	None	1.147 (0.710–1.853)
	**Adjusted**	1.471 (0.787–2.747)	1	1.487 (0.847–2.611)	1.058 (0.325–3.448)	None	0.920 (0.562–1.509)

The adjusted model includes gender, age, education, occupation, marriage status, living states, cognitive impairment family history, smoking, alcohol consumption, stroke, diabetes mellitus, heart disease, and hypertension. OR, odds ratio; 95%CI, 95% confidence interval; Ref, reference category; RBD, Rapid eye movement sleep behavior disorder; AD, Alzheimer’s disease; VaD, vascular dementia; DLB, dementia with Lewy bodies; ODs, other dementias. *p < 0.05, **p < 0.001.

Participants with RBD significantly showed 10 times higher risk of DLB than those without (*p* < 0.001). Moreover, hypersomnolence in the elderly population provided more opportunities to develop dementia (adjusted OR = 2.458, 95%CI: 1.519–3.978, *p* < 0.001), and particularly DLB (adjusted OR = 31.213, 95%CI: 17.618–55.301, *p* < 0.001).

**Figure 4:**
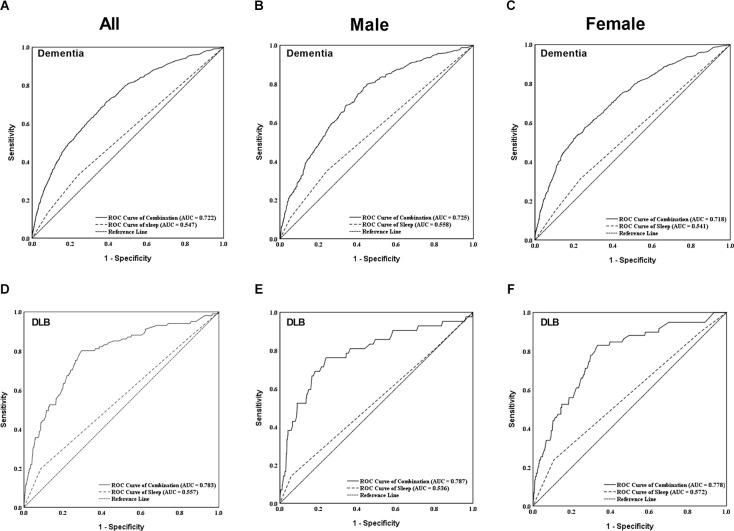
Figures showed the individual and combined diagnostic values of sleep duration for dementia **(A–C)** and DLB **(D–F)** in both males and females. The combined diagnostic values considered sleep duration, gender, age, education, marriage, living status, occupation, smoking, alcohol consumption, stroke, DM, heart disease, and hypertension mentioned at Table 1. DLB, dementia with Lewy bodies; ROC, receiver operating characteristic; AUC, area under the curve.

We generated a ROC curve to evaluate the combined diagnostic value of sleep duration for dementia and DLB (shown in Figure 4). The area under the curve (AUC) for the combination of sleep duration (<5 h and >8 h) were 0.722 (95%CI: 0.704–0.740, *p* < 0.001) and 0.783 (95% CI: 0.734–0.831, *p* < 0.001) for dementia and DLB, respectively. The results in the male and female populations were not significantly different.

## Discussion

In the present study of older adults, the prevalence rates of dementia and DLB were 12.2% and 1.3%, respectively. The <5 h or >8 h sleep duration, RBD and hypersomnolence had significant associations with occurrences of dementia and DLB. Furthermore, the percentage of the DLBs with the four individual core clinical features at the time of investigation was 44.54% for fluctuating cognition, 71.29% for visual hallucinations, 21.78% for RBD, and 27.71% for Parkinsonism. Our study provides a better understanding of associations between sleep characteristics and dementia in Chinese older adults and will help to improve the identification and the early diagnosis of DLB.

### Prevalence of DLB

Limited information is available about the incidence and prevalence of DLB in the general population. We found that the overall prevalence of DLB was 1.05% in individuals aged 65 years or older, which was similar to (Savica et al., 2013) or slightly higher than (Wada-Isoe et al., 2012) previous reports. In our study, the mean age of the participants was 74.85 years, with more females than males. The sex ratio of DLB is inconsistent. Scientific literature showed a traditional male predominance in DLB (Savica et al., 2013; Mouton et al., 2018), while a systemic review (Vann Jones and O’Brien, 2014) showed that five of eight studies reported disproportionately more women with DLB when controlling for the gender of the sample, and the other three reported disproportionately more men. Low education has been confirmed to be a risk factor for dementia, especially for AD and DLB (Xu et al., 2016; Yue et al., 2016). The average educational years of our participants (5.73 years) was lower than the participants in Japan (Wada-Isoe et al., 2012) and the United States (Savica et al., 2013), which might account for the inconsistency in prevalence. Moreover, socioeconomic, race, sample size, and study design might also contribute to the inconsistent results.

### Sleep and cognition

Short or long sleep duration had an association with CI in earlier epidemiological or experimental studies. In our study, a total of 9.1% of people sleeping for 7–8 h suffered from dementia, while the proportion showed a significant increasing trend along with the duration disturbances. Compared to participants with 5–8 h of sleep, those with <5 h were 1.299 times more likely to develop dementia, like those with >8 h of sleep. Our results were consistent with previous studies that showed that old-age long sleep duration (>8 h) was associated with an increased risk of incident dementia and proved that short sleep duration (<4 h) was associated with poorer cognitive function (Niu et al., 2016). The “glymphatic system” was shown to be responsible for cleaning the brain from waste products (such as Aβ and tau) during sleep, and it was strongly stimulated by sleep, where clearance during sleep was twofold faster than during waking hours (Mendelsohn and Larrick, 2013). The CABLE study (Xu et al., 2020) of 736 Chinese Han individuals suggested that insufficient or excessive sleep was associated with CSF indicators of greater cerebral amyloid deposition, including lower CSF Aβ42/Aβ40 ratio, higher CSF T-tau/Aβ42 ratio, and higher CSF P-tau/Aβ42.

DLB patients have a greater tendency to fall asleep at inappropriate times during the days, showing daytime drowsiness or lethargy and daytime sleep of ≥2 h. They also have more night-time sleep disturbances, including RBD (Boddy et al., 2007). Our study found an association between short sleep duration) <5 h) with increased risk of DLB (OR = 1.795, 95%CI: 1.055–3.054). Because of less research on sleep duration and DLB, the mechanisms behind sleep duration marking DLB have remained speculative. As DLB is the main type of alpha-synuclein disease, the reduced clearance of extracellular alpha-synuclein might be a potential pathway (Sohail et al., 2017). Meanwhile, Parkinsonism and RBD, as the core features of DLB, have a close association with abnormal sleep duration. The prospective population-based Rotterdam Study suggested that shorter sleep duration increased the risk of Parkinsonism, particularly PD (HR = 0.48, 95%CI: 0.23, 0.99) in the first 2 years of follow-up (Lysen et al., 2019). In contrast, it was reported that long sleep duration increased the risk for conversion to PD, as well as to RBD in the general population (Haba-Rubio et al., 2018). A neuropathological assessment of individuals without clinical PD examined the association of abnormal sleep and Lewy body pathology and found that greater sleep fragmentation increased the presence of Lewy body pathology and substantia nigra neuron loss, as well as demonstrating a higher odd of a pathological diagnosis of PD (Sohail et al., 2017). Other potential mechanisms underlying these findings are that metabolic disturbances and inflammation can contribute to AD pathophysiology. Sleep disruption can disturb the homeostasis and try to elevate blood glucose and blood lipid levels, or alter the glucose regulation through decreased cerebral glucose metabolism and alterations in endocrine function, then accelerate Aβ plaque formation, increase prevalence and aster progression of dementia (reviewed in Carroll and Macauley, 2019). It seems that exploring the exact relationship between sleep disorders, metabolic syndrome, and Aβ, tau or alpha-synuclein pathology will help to discover new pathogenic mechanisms and therapeutic targets in dementia. Moreover, sleep disorders impair the immune response and cause systemic inflammation by the dysregulation of substances such as TNF-α, IL-1β, and IL-6 (Franceschi et al., 2000; Atienza et al., 2018). The abnormal activation of microglia contributes to the impairment of hippocampal, frontal white matter, or blood-brain barrier permeability. Moreover, systemic inflammation can affect the clearance of Aβ, tau, or alpha-synuclein, leading to abnormal depositions and causing or exacerbating Parkinsonism and DLB.

### Characteristics of DLB at investigation

The current study was the first community-based investigation to explore the association between sleep and DLB in mainland China in recent years. We also described the clinical features that occur in participants with DLB.

We found a prevalence of DLB in this study of 1.30% in individuals aged 65 years or older, comprising 11.00% of overall dementia, which was slightly higher than our previous study reported in 2016 (*n* = 58, comprising 1.05% in all 5,542 participants and 10.36% in 574 participants with dementia; Yue et al., 2016). Our study confirmed that cognitive impairment is the most common symptom of DLB, with a higher frequency of visual hallucinations than those reported in previous clinical cases (Naasan et al., 2021). More than 80% of DLB patients showed fluctuating cognition in previous research (Walker et al., 2000), while we found a much lower frequency of fluctuating cognition in the Chinese DLB population in the current study. RBD and Parkinsonism were less likely to be evident. RBD is a core clinical feature, and REM sleep without atonia confirmed by polysomnography has been an important biomarker in the diagnosis of DLB (McKeith et al., 2020). RBD often begins many years before other symptoms and may become less vigorous or even quiescent over time, which led to inadequate unidentification of RBD in the current study. Compared with the DLBs who took the initiative to see the doctor, those diagnosed “passively” in this investigation were significantly older and had worse cognition performance (Gan et al., 2021). Participants with DLB experienced severe autonomic dysfunction (such as orthostatic hypotension, hyperhidrosis, constipation, urinary incontinence), repeated falls, hypersomnolence and other psychiatric symptoms such as delusions, hallucinations (except visual hallucinations), apathy, anxiety, and depression. As we had described the higher frequencies of visual hallucination and psychiatric symptoms might be associated with the impairment of anterior and posterior regions (secondary visual areas, orbitofrontal cortex, and anterior cingulate cortex; Liu et al., 2017). It also revealed a fact that older adults in Northern China had low awareness of DLB, which led to an irreversible consequence after being discovered. Prominent psychiatric symptoms and severe ANS dysfunction symptoms are related to high caregiver burden and poor quality of life of patients, respectively (Vernon et al., 2019). These disorders seriously disturb caregivers and patients, and are easier to be observed and noticed in daily life.

Our result was probably an accurate reflection of the frequencies of core and supportive clinical features in a DLB population of rural areas of China, though different from previous reports (Yue et al., 2016). The current study was the first community-based survey applying the fourth consensus report of the DLB Consortium in 2017 (McKeith et al., 2017). Moreover, a community-based study could prevent the subjective selection of patients. It might suggest that visual hallucination was the most recognizable symptom of DLB in the community. Therefore, our findings may help improve awareness and recognition of DLB in rural China.

### Limitations of the present study

First, because information on disease history or sleep characteristics were collected by self-report, participants with cognitive impairment and without caregivers as well, might not be able to accurately provide this information, and thus, we invited their friends or relatives to provide information about the participants to strengthen the study. Secondly, sleep disorders might be underestimated. Since the subjective sleep measurements might not completely reflect similar constructs as objective measurements (e.g., polysomnography, actigraphy), leading to an underestimation of RBD, which should be supplemented in a future study. Then, sleep apnea syndrome, restless leg syndrome, and other types of sleep disorders were not analyzed. In addition, participants with cognitive impairment are also predisposed to develop sleep disorders in their progress. Moreover, the carriers of dementia and DLB in our study were few, which may have limited us in exploring detaiedl characteristics. Though we held training and retraining for the senior medical students or medical staffs before and during the study to reduce bias, there was no measure of inter-rater reliability, which may still cause bias. Sleep-wake disorders have been reported to precede the diagnosis of Parkinsonism and other related synucleinopathies. However, as this was a cross-sectional study, knowing what came first (the sleep issue or the diagnosis of dementia/DLB) was not completely possible. An epidemiological survey of DLB focus on lower age groups, as well as prospective cohort studies to investigate associations with objective sleep and to assess the combined diagnostic value of short or long sleep, are needed in the future.

## Conclusions

Our findings provide a better understanding of the association between sleep characteristics and dementia in Chinese older adults and indicate the core clinical features of DLB among the general population. The prevalence of dementia was 12.2% in the participants, and 1.3% participants had DLB. We found that hypersomnolence and short (<5 h) or long (>8 h) sleep duration independently increased the risk of dementia, especially DLB. Moreover, visual hallucination (71.29%) was the most common core clinical feature of DLB at the time of investigation, followed by fluctuating cognition (44.54%), Parkinsonism (27.71%), and RBD (21.78%). Short or long sleep duration showed a combined diagnostic value for DLB.

## Data Availability Statement

The raw data supporting the conclusions of this article will be made available by the authors, without undue reservation.

## Ethics Statement

The studies involving human participants were reviewed and approved by the Committee for Medical Research Ethics at Tianjin Huanhu Hospital and the Tianjin Health Bureau (ID: 2019-40). The patients/participants provided their written informed consent to participate in this study.

## Author Contributions

YJ: conceptualization, project administration, and funding acquisition. YJ and BG: methodology. FW: software. ZS, YL, and YJ: validation. FW and ZC: formal analysis. JG, SL, YL, JN, XM, PC, and BG: investigation. ZS, X-DW, and YJ: resources. SL: data curation. JG: writing—original draft preparation. JG, ZS, X-DW, and BG: writing—review and editing. YL and JN: visualization. SL: supervision. All authors contributed to the article and approved the submitted version.

## Funding

This work was supported by the National Natural Science Foundation of China (grant number: 82171182), Science and Technology Project of Tianjin Municipal Health Committee (grant number ZC20121 and KJ20048), and Tianjin Key Medical Discipline (Specialty) Construction Project (grant number: TJYXZDXK-052B).
